# Maternal manipulation in the social Hymenoptera

**DOI:** 10.1073/pnas.2424729122

**Published:** 2025-01-21

**Authors:** Jacobus J. Boomsma

**Affiliations:** ^a^Department of Biology, University of Copenhagen, Copenhagen 2100, Denmark

Rees-Baylis et al. (1) claim that maternal underprovisioning can induce the evolution of reproductive altruism at reduced sibling relatedness. However, they modeled situations that have not occurred in nature, misinterpret the predictive domain of the monogamy hypothesis, and use the unfounded assumption that permanent castes arose from facultative reproductive role-differentiation.

The authors cite Hughes et al. (2) showing that the ancestors of the social bees, wasps and ants founded colonies via a single once inseminated female. Their models suggesting a polyandrous scenario should thus have prompted scrutiny of social insect biology, and of the likelihood that natural social evolution has been richer than a stated artificiality like: “[i]n our model, the mating frequency is fixed and cannot evolve. If the mating frequency was evolving, a plausible outcome would be that monandry evolves, and as a result, eusociality effectively always evolves in a monogamous population.” Indeed, no study has documented that polyandry is more than a fringe phenomenon among halictid, allodapine, and euglossine bees or stenogastrine and polistine wasps.

The authors maintain that the monogamy hypothesis (3) was developed to explain the evolution of eusociality. Although tests (2) and later versions (e.g., ref. 4) used that terminology, reading their own references should have clarified that the hypothesis only addressed the origin of preimaginal queen–worker caste differentiation in what William Morton Wheeler called superorganismal colonies (5)—only for those major transitions in level of organismality was strict monogamy conjectured to be a universal necessary condition (3, 6). Initially, the monogamy hypothesis was thus interpreted as predicting the origins of “obligate eusociality” until it appeared that conceptual clarity required dropping the eusociality term altogether (6, 7) in favor of distinguishing between 1) facultative, condition-dependent altruism in helper-societies [of both insects and vertebrates (8, 9)] and 2) obligate, unconditional altruism in superorganismal colonies [only in some insect lineages (3, 6)]. It was never argued that facultative altruism precludes some maternal manipulation in helper societies; only that obligate altruism cannot be established via maternal coercion.

The authors cite, but further ignore, the review concluding that broad-brush eusociality sensu Wilson (10) has been a root cause of confusion because it is not a trait that can respond to selection (7), in contrast to facultative and obligate altruism. Eusociality terminology forces reality into a preordained artificial human construct that precludes recognizing that helper societies and caste-differentiated lineages evolved independently from solitary or very weakly social ancestry (6). The authors only modeled helpers in populations of halictid bees but then erroneously extrapolated their approach to be relevant for preimaginal caste differentiation, which never evolved in halictids. They use hand-waving language like “has been suggested,” “could alleviate,” and “could favor,” hiding the fact that there is no empirical evidence for such “eusociality-gradient” transitions having ever happened in the Hymenoptera.

Models can be useful tools, but only when the assumptions are as realistic as the predictions. Without such care, models are misleading, particularly when they claim to illuminate evolutionary directions that nature has never taken.
